# Efficacy of Chlorogenic Acid Combined With Cefazolin Against Methicillin-Resistant *Staphylococcus aureus* Biofilms

**DOI:** 10.1155/cjid/6755742

**Published:** 2025-06-26

**Authors:** Borel Ndezo Bisso, Humera Jahan, Jean Paul Dzoyem, M. Iqbal Choudhary

**Affiliations:** ^1^Department of Biochemistry, Faculty of Science, University of Dschang, Dschang 1499, Cameroon; ^2^Dr. Panjwani Center for Molecular Medicine and Drug Research, International Center for Chemical and Biological Sciences, University of Karachi, Karachi 75270, Pakistan; ^3^Institute of Molecular Biology and Biotechnology, The University of Lahore, Lahore 54000, Pakistan; ^4^H.E.J. Research Institute of Chemistry, International Center for Chemical and Biological Sciences, University of Karachi, Karachi 75270, Pakistan; ^5^Department of Chemistry, Faculty of Science and Technology, Universitas Airlangga, Komplek Kampus C, Jl. Mulyorejo, Surabaya 60115, Indonesia

## Abstract

**Background:** Methicillin-resistant *Staphylococcus aureus* (MRSA), has the ability to cause biofilm associated chronic infections with a high mortality rate. This creates a demand for the improved antibiofilm therapies. The aim of this study was to evaluate the anti-MRSA and antibiofilm activity of a natural product, chlorogenic acid, in synergy with an antibiotic, cefazolin.

**Material and Methods:** The synergistic effect was measured by the checkerboard method. The antibiofilm activity was analysed by crystal violet staining, MTT assay, and atomic force microscopy (AFM). The cytotoxic effect on Human Embryonic Kidney (HEK 293) cells was determined using the MTT assay. Assays were performed in triplicate, and compared using the one-way ANOVA test.

**Results:** When chlorogenic acid and cefazolin were combined at low concentrations, a strong biofilm inhibition in terms of biofilm biomass (88%), and metabolic activity (82%) was observed, as compared to the results obtained for each compound alone. AFM images of biofilms, treated with chlorogenic acid combined with cefazolin, revealed a high destruction of biofilms and extracellular polymeric substances, as compared to each drug alone. A nontoxic effect on HEK 293 cells was observed for the combination of chlorogenic acid and cefazolin.

**Conclusion:** Chlorogenic acid can be used as an adjuvant with currently used antibiotic in the development of combinatory therapies to treat biofilm-associated bacterial infections.

## 1. Introduction

Biofilms are communities of microorganisms that adhere to a surface and surrounded by a self-produced matrix, composed of exopolysaccharides, proteins, and nucleic acids [1, 2]. The biofilm matrix protects microbes against the host immune system and antimicrobial agents, thus causing chronic and resistant infections. Approximately 80% of bacterial infections are due to the ability of pathogens to form biofilms. Biofilms are 1000 times more resistant to conventional antibiotics than planktonic cells [3, 4]. This is due to the limited penetration of antimicrobial agents into the biofilm matrix, and the nutrient, and oxygen gradients, as well as horizontal transfer of resistance genes of biofilm colonies [5, 6].

According to the World Health Organization (WHO), methicillin-resistant *Staphylococcus aureus* (MRSA) belongs to the list of priority pathogens [7]. MRSA causes many infections, such as pneumonia, bloodstream infections, and surgical site infections that are associated to high morbidity and mortality rates [8, 9]. Recent decades have been marked with the emergence and spread of multidrug-resistant MRSA strains with an increase in MRSA infections from 7% to 60% between 1964 and 2015. However, this increasing antibiotic resistance in MRSA strains has been due to their ability to form biofilms on host mucosa and medical devices, thus limiting treatment [10, 11]. Although vancomycin remains the most widely used antibiotic to treat MRSA infections, there are not many therapeutic options to treat biofilm-related infections caused by MRSA [12–14]. Therefore, it is increasingly important to identity new antimicrobial agents against MRSA, and its biofilms.

In the clinical practice, antibiotic combinations are frequently used to combat microbial resistance. In fact, antibiotic combinations can reduce resistance, treat recalcitrant infections, reduce drug toxicity, and improve drug effectiveness [15–18]. In this regard, several combinations of commercial antibiotics with bioactive natural products were used for the treatment of bacterial infections. Furthermore, the synergistic effect of antimicrobial agents was found more effective than that of a single antimicrobial agent [19–22]. Therefore, we hypothesize that plant-derived natural products could augment the efficacy of conventional antimicrobial agents in the treatment of biofilm-related infections.

Chlorogenic acid is a naturally occurring bioactive polyphenol found abundantly in *Gardenia jasminoides* Ellis. Recently, some studies have reported its biological activities, such as antioxidant, antitumor, anti-inflammatory, and protection of the liver and kidneys [23, 24]. Chlorogenic acid has also demonstrated antibacterial activity against Gram-positive and Gram-negative bacteria by inhibiting bacterial cell membrane synthesis, interfering with normal cell cycle progression, and disrupting bacterial cell metabolism of bacterial cells [25, 26]. In addition, chlorogenic acid inhibits biofilm formation, and acts synergistically with levofloxacin against *Klebsiella pneumoniae* biofilms [27]. However, studies on its synergistic antibiofilm effect with antibiotic against MRSA are scarce. The aim of the present study was to investigate the synergistic effect of chlorogenic acid and cefazolin against MRSA biofilms.

## 2. Materials and Methods

### 2.1. Bacterial Strain and Reagents

MRSA NCTC 13277 was obtained from the Microbial Bank of the Dr. Panjwani Center for Molecular Medicine and Drug Research at the International Center for Chemical and Biological Sciences (ICCBS), University of Karachi, Pakistan, and kept in tryptic soy broth (TSB) with 20% glycerol, and stored at −80°C. Before use, the strain was grown in TSB at 35°C for 24 h. Dimethyl sulfoxide (DMSO), crystal violet, cefazolin, chlorogenic acid (purity ≥ 98%), and 3-(4, 5-dimethyl-2-thiazolyl)-2,5-diphenyl-2H-tetrazolium-bromide (MTT) were purchased from Sigma-Aldrich (Germany).

### 2.2. Determination of Minimum Inhibitory Concentration (MIC) and Minimum Bactericidal Concentration (MBC)

The MICs and MBCs of chlorogenic acid and cefazolin were determined by using the broth microdilution method [28]. Serial two-fold dilutions of chlorogenic acid and cefazolin in concentrations ranging from 256 to 0.125 μg/mL with bacterial inoculum (1 × 10^8^ CFU/mL) was added in a 96-well microplate, followed by incubation at 37°C for 24 h. The negative control contained only broth, while the positive control contained broth plus bacteria. The absorbance was measured at 600 nm using a spectrophotometer (MultiSkan Go, Thermo Scientific, USA). The MIC was defined as the lowest concentration of chlorogenic acid and cefazolin that inhibits the visible growth of bacteria. According to the cutoff values of the Clinical Laboratory Standard Institute (CLSI), *S. aureus* is resistant to cefazolin when MIC ≥ 16 μg/mL [29]. The MBC was determined by subculturing 50 μL from the wells that did not showed visible bacterial growth on agar plate at 37°C for 48 h. The MBC was defined as the lowest concentrations of chlorogenic acid and cefazolin that kills 100% of bacteria [30].

### 2.3. Antibiofilm Assay

The minimum biofilm inhibitory concentration (MBIC) and minimum biofilm eradication concentration (MBEC) of chlorogenic acid and cefazolin were determined, as previously described [31]. In brief, 100 μL of serial two-fold dilutions of chlorogenic acid and cefazolin at concentrations of 1/2, 1/4, 1/8, and 1/16 MIC were transferred into each well of the 96-well flat-bottomed polystyrene microplate, followed by the addition of 100 μL of bacterial inoculum (1 × 10^8^ CFU/mL). The microplate was incubated at 37°C for 24 h. The microplate was then discarded and washed with phosphate-buffered saline (PBS) to remove the nonadherent cells. The biofilms were stained with 200 μL of 0.1% crystal violet solution at room temperature for 20 min. After incubation, the excess of crystal violet was removed, and 200 μL of 33% glacial acetic acid was added to dissolve the dye. To quantify the biofilm biomass, the absorbance at a wavelength of 570 nm was measured using a microplate reader (MultiSkan Go, Thermo Scientific, USA). The percentage of biofilm inhibition was calculated as follows:

(1) $$\begin{array}{c} \%\text{ Biofilm inhibition}=\left[1-\left\{\frac{\left(\text{ODtest}-\text{ODblank}\right)}{\left(\text{ODcontrol}-\text{ODblank}\right)}\right\}\right]\times 100. \end{array}$$

MBIC was defined as the lowest concentration of chlorogenic acid or cefazolin inhibiting at least 80% of biofilm biomass.

For the determination of MBEC, 100 μL of bacterial inoculum (1 × 10^8^ CFU/mL) was transferred into each well of the 96-well flat-bottomed polystyrene microplate, followed by incubation at 37°C for 24 h. The plate was then discarded, and the biofilms were treated with serial two-fold dilutions of chlorogenic acid and cefazolin at concentrations of 2 × MIC, 4 × MIC, 8 × MIC, and 16 × MIC. After incubation at 37°C for 24 h, the plate was treated, as described above. The percentage of biofilm eradication was calculated using the same formula for the calculation of the percentage of biofilm inhibition. MBEC was defined as the lowest concentration of chlorogenic acid or cefazolin eradicating at least 80% of biofilm biomass.

### 2.4. Cell Viability of Biofilm

The effect of chlorogenic acid and cefazolin on biofilm metabolic activity was determined by MTT assay [32]. In brief, after treating the biofilms with chlorogenic acid and cefazolin as described above, the plate was discarded and 200 μL of MTT solution (0.5 mg/mL) was transferred into each well of the 96-well flat-bottomed polystyrene microplate, followed by incubation for 3 h in the dark at 37°C. Then, the plate was discarded and the purple formazan that formed inside the bacterial cells was dissolved by adding 100 μL of DMSO, followed by measuring the absorbance at a wavelength of 570 nm using a microplate reader (MultiSkan Go, Thermo Scientific, USA).

### 2.5. Checkerboard Microdilution Assay

The antibiofilm effect of chlorogenic acid, combined with cefazolin, against biofilm inhibition and eradication of mature biofilms was assessed by the checkerboard method [32]. Two-fold serial dilutions of chlorogenic acid (1024–16 μg/mL), and cefazolin (1024–8 μg/mL) were added to the rows and columns of the plates, respectively. Then, 100 μL of bacterial inoculum (1 × 10^8^ CFU/mL) was transferred into each well of the microplate. After incubation at 37°C for 24 h, biofilm biomass and metabolic activity were determined as described above.

To investigate the synergistic effect of chlorogenic acid with cefazolin against the eradication of mature biofilms, biofilms were preformed into the wells of the 96-well flat-bottomed polystyrene microplate and then treated, as described above. The MBIC and MBEC of antimicrobial agents alone and in combination were determined as mentioned above. Drug interactions were interpreted on the basis of the fractional inhibitory concentration index (FICI). FICI was defined as the sum of FIC_(chlorogenic acid)_ and FIC_(cefazolin)_, where FIC of each antimicrobial agent is recorded as the MBIC or MBEC of each antimicrobial agent used in combination divided by MBIC or MBEC of each antimicrobial agent used alone. FICI values were interpreted as follows: synergy, FICI ≤ 0.5; additive, 0.5 < FICI ≤  1; indifferent, 0.5 < FICI  ≤ 4.0; and antagonism, FICI  > 4.0 [32].

### 2.6. Atomic Force Microscopy (AFM) Analyses of Biofilms

Morphological analyses of inhibition of biofilm formation and eradication of mature biofilm, induced by chlorogenic acid and cefazolin alone and their combination, were analyzed by AFM. For the morphological analysis of biofilm inhibition, 24-well plate containing silicon chips (5 × 5 mm) was filled with *S. aureus* cells (1 × 10^6^ CFU/mL) and chlorogenic/cefazolin alone and their combination. After incubation at 37°C for 24 h, the silicon chips were washed with PBS to remove nonadherent cells, and the biofilms were visualized by AFM (Agilent Technologies 5500, USA). For morphological analysis of eradication of mature biofilms, biofilms were placed on silicon chips (5 × 5 mm) and then treated with chlorogenic/cefazolin alone and their combination as described above, following their observation using AFM (Agilent Technologies 5500, USA).

### 2.7. Cytotoxicity Studies of a Synergistic Combination of Chlorogenic Acid and Cefazolin

The cytotoxicity of the combination of chlorogenic acid and cefazolin was evaluated on human embryonic kidney (HEK 293) cells through MTT assay [12]. In brief, HEK 293 cells at concentration of 5 × 10^4^ cells/well prepared in culture medium (Dulbecco's modified eagle medium + 10% of fetal bovine serum) were seeded into 96-well microplate. After incubation for 24 h at 37°C in a 5% CO_2_, the medium was gently removed and HEK 293 cells were treated, as described above, and allowed to incubate for 24 h at 37°C in a 5% CO_2_. Then, MTT solution (100 μL/well, 5 mg/mL) was added, followed by incubation in the dark for 4 h. DMSO (100 μL/well) was added to dissolve the purple formazan crystals to pink color and the absorbance was measured at 570 nm. The percentage of cell viability was calculated as follows:

(2) $$\begin{array}{c} \%\text{ Cell viability}=\left[\frac{\left(\text{ODtest}-\text{ODblank}\right)}{\left(\text{ODcontrol}-\text{ODblank}\right)}\right]\times 100. \end{array}$$

### 2.8. Statistical Analysis

Results are reported as the mean ± standard deviation (SD) (*n* = 3) and analyzed by the one-way ANOVA test by GraphPad prism Version 9.5.1. *p* < 0.05 were considered statistically significant.

## 3. Results

### 3.1. Antibacterial and Antibiofilm Activities

Chlorogenic acid and cefazolin inhibited the growth of MRSA at the concentrations of 256 and 128 μg/mL, respectively. The biofilm formation of MRSA was inhibited by subinhibitory concentrations of chlorogenic acid (128 μg/mL) and cefazolin (64 μg/mL). However, high concentrations of chlorogenic acid (1024 μg/mL) and cefazolin (512 μg/mL) were needed to destroy the mature biofilms of MRSA (Table 1).

### 3.2. Synergistic Antibiofilm Activity

**Figure 1:**
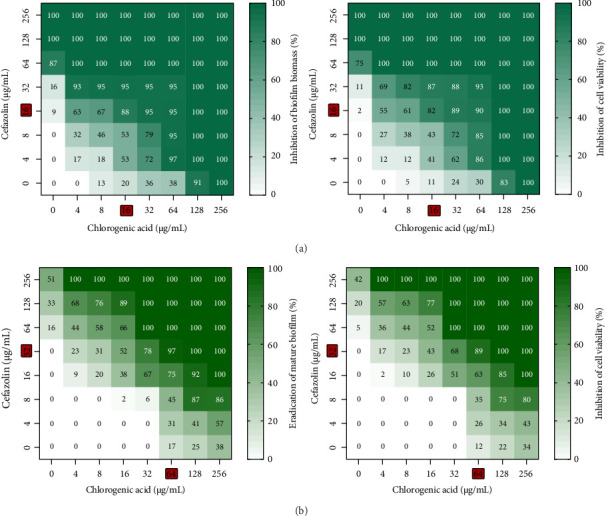
Effect of individual and combinatorial treatments of chlorogenic acid and cefazolin against biofilm formation and mature biofilms in MRSA. (a) Effect of treatments on the inhibition of biofilm formation. (b) Effect of treatments on the eradication of mature biofilm. The concentrations of drug that shows synergistic effect were represented in red color.

The checkerboard method revealed synergistic effects when chlorogenic acid was combined with cefazolin against biofilm formation and eradication of mature biofilm in MRSA (Table 2). The percentage of inhibition and eradication of the biofilm, as well as the percentage of inhibition of biofilm metabolic activity by chlorogenic acid and cefazolin alone, as well as their combination, is shown in Figure 1. The combination (16 μg/mL cefazolin + 16 μg/mL chlorogenic acid) significantly prevented the biofilm formation, reducing biofilm biomass and cell viability by 88% and 82%, respectively. A strong reduction in biofilm biomass and metabolic activity of 97% and 89%, respectively, was observed when chlorogenic acid (64 μg/mL) was combined with cefazolin (32 μg/mL).

### 3.3. AFM Analyses

**Figure 2:**
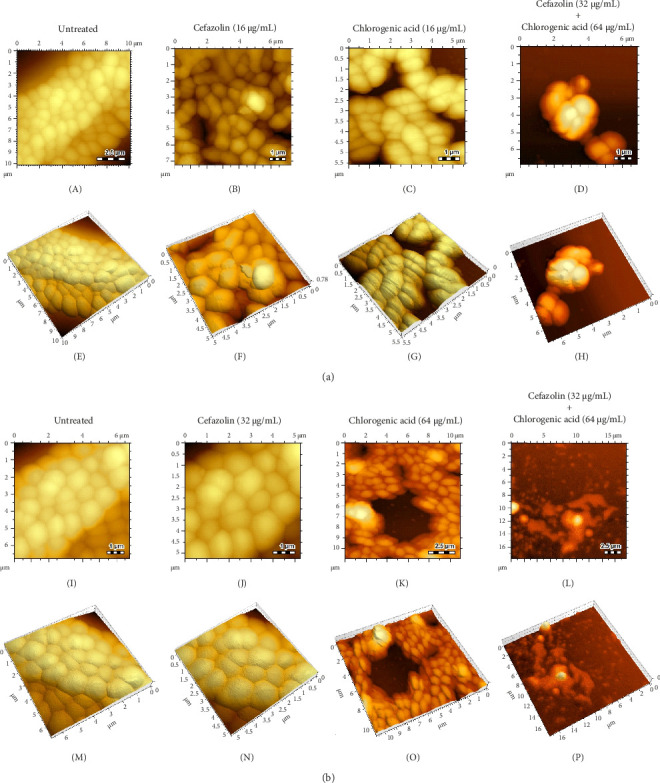
AFM analyses of the synergistic antibiofilm effect of chlorogenic acid and cefazolin against MRSA. (a) AFM images of the combination of chlorogenic acid and cefazolin against biofilm inhibition and (b) AFM images of the combination of chlorogenic acid and cefazolin against eradication of mature biofilm: (A–D; I–L) represents the AFM 2D topography images and (E–H; M–P) represents the AFM 3D topography images.

Figure 2 shows the AFM images of the combination effect of chlorogenic acid and cefazolin on MRSA biofilms. The untreated biofilms showed dense cells covered by extracellular polymeric substances. Regarding the inhibition of biofilm formation, AFM images of biofilms treated with chlorogenic acid or cefazolin alone at a concentration of 16 μg/mL showed that biofilm formation was weakly inhibited, as compared with untreated biofilms. However, when chlorogenic acid was combined to cefazolin at the same concentration (16 μg/mL), the AFM images revealed a higher inhibition of biofilm formation, characterized by a small number of adherent cells.

Regarding the eradication of mature biofilms, there was no difference in AFM images of untreated biofilms and biofilms treated with cefazolin at a concentration of 32 μg/mL. The AFM image of biofilms, treated with chlorogenic acid at a concentration of 64 μg/mL, showed the destruction of biofilms. In contrast to untreated biofilms, AFM image of biofilms treated with chlorogenic acid combined with cefazolin revealed a high destruction of biofilms and extracellular polymeric substances, as compared to each drug.

### 3.4. Cytotoxicity

**Figure 3:**
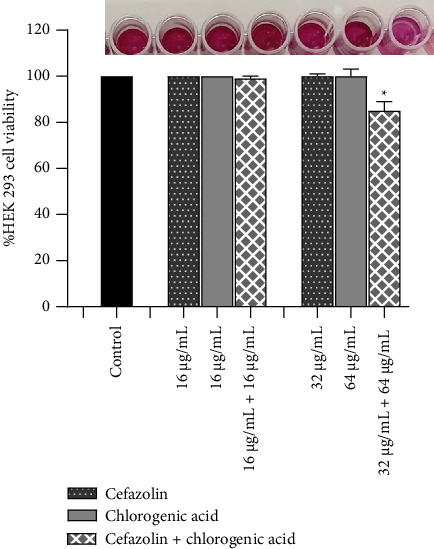
Cytotoxicity effect of chlorogenic acid and cefazolin alone, and their combination on HEK 293 cells. ^∗^*p* < 0.05: statistically significant difference, as compared to the untreated control.

HEK 293 cells treated with chlorogenic acid and cefazolin alone at the same concentration (16 μg/mL) and their combination (16 μg/mL cefazolin + 16 μg/mL chlorogenic acid) displayed the same percentage of cell viability of 100%. When HEK 293 cells were exposed to high concentrations of cefazolin (32 μg/mL) and chlorogenic acid (64 μg/mL), the percentage of cell viability was also 100%. However, the combination (32 μg/mL cefazolin + 64 μg/mL chlorogenic acid) showed a percentage of HEK 293 viable cells of 85% (Figure 3).

## 4. Discussion

Bioactive natural products are promising antimicrobial agents to combat antibiotic resistance [31, 33, 34]. In this study, natural product chlorogenic acid, demonstrated antibacterial activity against MRSA. Apparently, chlorogenic acid exerts its antibacterial activity through several mechanisms, such as inhibition of synthesis of bacterial cell membranes, and disruption of cell metabolism of bacterial cells [25, 26].

In this study, we have also investigated the antibiofilm activity of chlorogenic acid against MRSA. Our results showed that chlorogenic acid prevented the biofilm formation at subinhibitory concentration. This could be explained by its ability to disrupt cell-surface interaction and interfere with bacterial adhesion that promote bacterial adhesion to the surface, thereby reducing bacterial cell adhesion [35]. However, high concentrations of chlorogenic acid and cefazolin were required to destroy the mature biofilms of MRSA. These results could be attributed to the structural composition of the biofilm. In fact, extracellular polysaccharide substances that surround the biofilm cells serve as a protective barrier against host immune system and drugs [6]. The low availability of oxygen and the nutrient in the deeper layers of the biofilm lead to cellular dormancy, making biofilm cells nonsusceptible to antibiotics like β-lactams [36]. Furthermore, the close contact of cells in the biofilm promotes horizontal gene transfer, leading to the spread of antibiotic resistance genes [37]. It is, therefore, necessary to develop new antimicrobial strategies to combat the biofilm-related infections.

Drug combinations are frequently used in medicine to overcome antibiotic resistance. In this study, we evaluated the efficacy of chlorogenic acid, combined with cefazolin, to prevent and eradicate MRSA biofilms. Synergistic effects were observed when chlorogenic acid was combined with cefazolin against both inhibition of biofilm formation and eradication of mature biofilm. Many studies have reported the synergistic effect of chlorogenic acid and antibiotics against bacterial pathogens, such as *K. pneumoniae*, *Escherichia coli*, and *Listeria monocytogenes* [27, 38, 39]. The synergistic effect of chlorogenic acid and cefazolin considerably decreased the biofilm biomass and metabolic activity of biofilm colony. This reduction in biomass and metabolic activity of biofilm cells are the much needed characteristics of effective antibiotics, capable of disrupting the biofilm structure [1].

Powerful and diverse microscopic tools are necessary to study antibiofilm activity. AFM appears as an excellent microscopic method for studying microorganisms, as well as the antimicrobial effect of drugs against microorganisms [40]. AFM provides microbial topography at nanometer resolution, allowing the detection of changes in their morphology and abnormalities in their structures, after drug treatment, as well as to understand the mechanisms of action of drugs [41]. Therefore, we used the AFM to observe and understand the effect of combination of chlorogenic acid and cefazolin in *S. aureus* biofilms. AFM images showed that treatment with the combination of chlorogenic acid and cefazolin strongly prevented the biofilm formation and disrupted preformed biofilms, as compared to the individual drug or untreated cells. These results are similar to those obtained by analytical methods, and clearly confirm the combined effect of chlorogenic acid and cefazolin on MRSA biofilms.

Cytotoxicity evaluation represents an important factor in the development of antimicrobial therapy. The HEK-293 are the normal human cells widely used in biological research due to their ability to grow without serum, rapid growth, and reliability [42]. Analysis of the metabolic activity of the HEK-293 cell by MTT assay makes it possible to elucidate the toxicity of a potential drug [43]. Therefore, we evaluated the cytotoxicity of the combination of chlorogenic acid and cefazolin by performing cell viability assay on HEK 293 cells using the MTT assay. It was observed that the percentage of viability HEK 293 cells after treatment with chlorogenic acid, combined with cefazolin, was greater than 80%. According to ISO 10993-5, there is a noncytotoxic effect when cell viability percentage is > 70% [44]. Hence, we can conclude that the combination of chlorogenic acid and cefazolin is noncytotoxic.

## 5. Conclusion

The current study demonstrated that natural product chlorogenic acid exhibited antibacterial and antibiofilm activities against MRSA. The cell viability of *S. aureus* was significantly reduced by the combination of chlorogenic acid and cefazolin. Chlorogenic acid synergistically enhanced the activity of cefazolin against MRSA biofilms. The combination of chlorogenic acid and cefazolin was noncytotoxic. Their combination, and possibly combination of chlorogenic acid with other conventional antibiotics can serve as potential alternative approach for the treatment of biofilm-associated infections. In future studies, we will perform the molecular evaluation of biofilm genes inhibited by the combination of chlorogenic acid and cefazolin, and investigate the effect of these combinations loaded into nanocarriers for the development of novel antibiofilm drugs.

## Figures and Tables

**Table 1:** Minimum inhibitory concentration, minimum bactericidal concentration, minimal biofilm inhibitory concentrations, and minimal biofilm eradication concentrations values (μg/mL) of chlorogenic acid and cefazolin against MRSA.

		*S. aureus* NCTC 13277
Chlorogenic acid	MIC	256
	MBC	1024
	MBIC	128
	MBEC	1024

Cefazolin	MIC	128
	MBC	256
	MBIC	64
	MBEC	512

Abbreviations: MBC = minimum bactericidal concentration (μg/mL), MBEC = minimal biofilm eradication concentration (μg/mL), MBIC = minimal biofilm inhibitory concentration (μg/mL), MIC = minimum inhibitory concentration (μg/mL).

**Table 2:** Checkerboard results of the combination of chlorogenic acid and cefazolin against MRSA biofilms.

	**MBIC alone**		**MBIC in combination**		**FICI**	**Interaction**
	**Chl**	**Cef**	**Chl**	**Cef**		

*S. aureus* NCTC 13277	128	64	16	16	0.375	Synergy

	**MBEC alone**		**MBEC in combination**			
	**Chl**	**Cef**	**Chl**	**Cef**		

*S. aureus* NCTC 13277	1024	512	32	64	0.156	Synergy

*Note:* Chl: chlorogenic acid, Cef: cefazolin.

Abbreviations: FICI = fractional inhibitory concentration index, MBEC = minimal biofilm eradication concentration (μg/mL), MBIC = minimal biofilm inhibitory concentration (μg/mL).

## Data Availability

The data used to support the findings of this study are available from the corresponding authors upon reasonable request.
